# Clinical Outcomes of Intravesical Prostatic Protrusion in Patients With Benign Prostatic Hyperplasia

**DOI:** 10.7759/cureus.52541

**Published:** 2024-01-19

**Authors:** Ahmed A Al Rashed, Qasim M Isa, Amina Mahdi, Mohamed Ebrahim, Khalid Abdulaziz, Omran Hasan, Basma D Malalla, Abdolsalam Ahmadi, Nader Awad

**Affiliations:** 1 Urology, Salmaniya Medical Complex, Manama, BHR; 2 Radiology, Salmaniya Medical Complex, Manama, BHR

**Keywords:** lower urinary tract symptoms, acute urinary retention (aur), transabdominal ultrasound, intravesical prostatic protrusion, prostate enlargement

## Abstract

Background

Benign prostatic hyperplasia (BPH) is a disorder that is characterized by the hyperplasia of the cellular elements of the prostate, leading to an enlarged prostate. One of the parameters affecting urinary outflow is intravesical prostatic protrusion (IPP). It is a phenomenon wherein the enlargement of the prostate protrudes into the bladder along the plane of least resistance. This condition can lead to various clinical effects, including symptoms such as the feeling of incomplete void and weak, interrupted urine stream. Hence, investigating the potential associations between different grades of IPP and clinical urological outcomes holds crucial implications for optimizing patient care, refining risk stratification, and enhancing treatment approaches.

Methodology

We examined patients who were following up at the urology outpatient clinics due to BPH between June 1, 2021, and December 31, 2022. All patients included in this study were required to undergo a transabdominal prostate ultrasound. Patient records were reviewed for various factors, including demographic stratification, the presence of urine routine or culture with evidence of urinary tract infection (UTI) within the past two years, and whether patients were scheduled for surgical intervention. The radiological parameters were recorded by viewing the midsagittal and transverse ultrasound images retrospectively by two specialist radiology physicians. The parameters measured included IPP Grade, prostate volume (PV), presence of bladder stones, anatomical abnormalities (such as bladder diverticulum), and post-void volume.

Results

The total sample size was 184 patients. Out of these, 53 (28.8%) had IPP Grade I, 72 (39.1%) were classified as Grade II, 42 (22.8%) had Grade III, and 17 (9.2%) were categorized as Grade IV. The data collected also showed that 12 (6.5%) patients had bladder stones on ultrasound examination. Additionally, 17 (9.2%) patients had bladder diverticulum. Furthermore, when controlled for age and PV, multivariate analysis using logistic regression models to calculate the odds ratio (OR) showed that increasing IPP Grade is associated with an increased risk of developing UTIs, acute urinary retention, and the need for surgical intervention. The highest risk group of patients is IPP Grade IV, with odds ratios (ORs) of 6.8, 7.2, and 6.4 for developing UTIs, experiencing acute urinary retention, and requiring surgical intervention, respectively.

Conclusions

The results provide compelling evidence of the adverse relationships between higher grades of IPP and worsening urological outcomes and patient morbidity. Hence, we recommend further studies be conducted on the clinical effects of IPP and that these measurements should be considered as part of routine ultrasound prostate imaging to aid in the management of BPH cases.

## Introduction

Benign prostatic hyperplasia (BPH) is a disorder characterized by the hyperplasia of the cellular elements of the prostate, leading to an enlarged prostate gland [1]. It is considered one of the most widespread diseases among men, with incidence increasing progressively with age, potentially leading to a progressive obstruction of urine flow [1]. The prevalence and impact of BPH on male urinary health have prompted extensive research aimed at understanding its etiology, progression, and associated clinical implications. However, there is currently no consensus to determine the severity of this obstruction, other than pressure-flow studies [2].

One of the parameters affecting urinary outflow is intravesical prostatic protrusion (IPP). It is a phenomenon wherein the enlargement of the prostate protrudes into the bladder along the plane of least resistance [2]. The enlargement seen in IPP can primarily result from the prostatic median lobe. However, tri-lobular enlargement is also capable of occupying the bladder space to a measurable capacity, thereby causing outflow obstruction [2,3]. The degree of IPP is generally measured vertically from the maximum tip (peak) of the intravesical protrusion to the circumference of the bladder at the bladder neck. The measurement is graded based on distance in millimeters, and the classification is as follows: Grade I (<5 mm), Grade II (5-10 mm), Grade III (10-15 mm), and Grade IV (>15 mm) [4].

This condition can lead to various clinical effects, including symptoms such as increased urinary frequency, urgency, and difficulty in starting or maintaining a steady stream of urine [5]. Additionally, IPP may cause incomplete bladder emptying, leading to high residual urine volumes within the bladder, which, in turn, can increase the risk of urinary tract infections (UTIs) and bladder stone formation [5]. Management of the clinical manifestations of BPH and high grades of IPP typically involves addressing the underlying causes and may include medications, minimally invasive procedures, or surgical intervention, depending on the severity and impact of the condition on the patient's quality of life [5].

The significance of studying the clinical effects of IPP is underscored by the substantial burden posed by BPH-related complications, including UTIs, acute urinary retention, and the potential need for surgical intervention [6]. These adverse events not only impact the quality of life for affected individuals but also place significant demands on healthcare systems and resources [6]. Therefore, investigating the potential associations between different grades of IPP and these urological outcomes is not merely an academic pursuit; rather, it holds crucial implications for optimizing patient care, refining risk stratification, and enhancing treatment approaches.

The present-day medical landscape favors an individualized approach to patient care, advocating for tailored interventions that consider the specific characteristics and prognosis of each patient [6-7]. In this context, the potential identification of prostatic protrusion grades as predictive factors for adverse urological events could mark a substantial advancement in the personalized management of BPH-related complications. By recognizing the prognostic value of IPP grading, our study’s primary aim is to explore the impact of IPP Grades on the development of UTIs, acute urinary retention, and the likelihood of requiring surgical intervention.

## Materials and methods

In this study, we examined patients who were following up with the urology outpatient clinics for obstructive lower urinary tract symptoms due to BPH between June 1, 2021, and December 31, 2022. All patients included in this study must have undergone a transabdominal prostate ultrasound. Patient records were examined for various factors, including demographic stratification, the presence of urine routine or culture with evidence of UTI within the past two years, and whether patients were scheduled for surgical intervention. In our institute, the two major surgeries performed for BPH are transurethral resection of the prostate (TURP) for prostate sizes less than 100 g and open prostatectomy for those exceeding 100 g. The need for surgical intervention is indicated in our institute for patients who typically have one of the following parameters: at least one failed trial without a catheter following an episode of acute urine retention or chronic complications such as bladder diverticulum, bladder stones, high post-void residual volume, recurrent hematuria, recurrent UTI, or renal function impairment. Patients with a history of prior prostatic or pelvic surgery, confirmed diagnoses of prostate or urothelial cancer, a history of ureteral strictures, and those in whom ultrasound studies proved inconclusive, leading to a poorly visualized prostate, were excluded from the study. 

The radiological parameters were recorded by viewing the midsagittal and transverse ultrasound images retrospectively by two specialist radiology physicians. The ultrasound device used was a GE LOGIQ E9 ultrasound machine (General Electric, Boston, MA) using a curvilinear 5-7.5 Hz transducer probe. The parameters measured included IPP Grade, prostate volume (PV), presence of bladder stones, anatomical abnormalities (such as bladder diverticulum), and post-void volume.

To provide a comprehensive overview of our patient cohort, measures such as mean, median, standard deviation, and frequency distributions were used to present the distribution of these variables. To assess the associations between prostatic protrusion grades and urological outcomes, bivariate analyses were performed. Specifically, chi-square tests were employed to examine the relationships between the categorical variable of prostatic protrusion grade and the occurrences of UTIs, acute urinary retention, and surgical requirements. These analyses were used to assess whether there were significant associations between the grade of prostatic protrusion and the prevalence of each urological outcome.

Furthermore, variables such as age, PV, and other relevant clinical factors were included as covariates in logistic regression models to assess whether the observed associations between prostatic protrusion grades and urological outcomes remained significant after accounting for other influential factors. This facilitated the establishment of whether the severity of prostatic protrusion independently impacted the risk of UTIs, acute urinary retention, and the need for surgery. All statistical analysis was conducted using IBM SPSS Statistics for Windows, Version 24.0 (IBM Corp., Armonk, NY), and *P*-values of <0.05 were considered statistically significant.

## Results

A total of 216 patients were referred from the urology outpatient department for a Transabdominal Ultrasound (TUS) during the study period. Thirty-two of these patients were excluded from the study based on the mentioned exclusion criteria, resulting in a total sample size of 184 patients. Among these, 53 patients (28.8%) had IPP Grade I, 72 patients (39.1%) had Grade II, 42 patients (22.8%) had Grade III, and 17 patients (9.2%) had Grade IV. The demographics of the study's sample size are presented in Table 1.

**Table 1:** Demographic variables. IPP, intravesical prostatic protrusion; SD, standard deviation

Variable	Average ± SD	Maximum	Minimum
Age (years)	62.5 ± 7.2	90	42
Prostate volume (mL)	46.1 ± 15.5	160	12
IPP (mm)	9.85 ± 2.4	31	2

**Figure 1:**
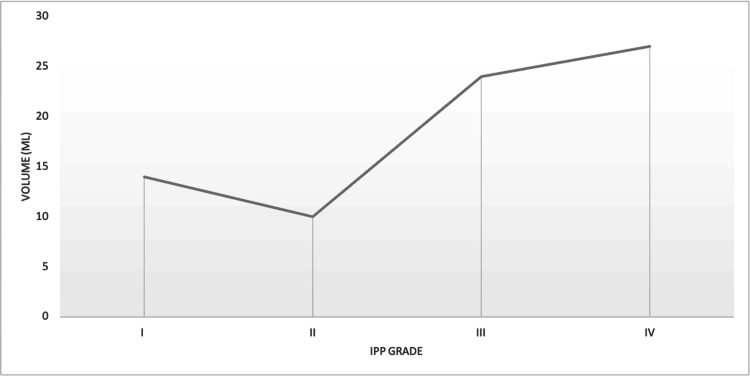
Mean post-void volume (in mL) for each IPP Grade. IPP, intravesical prostatic protrusion

The data collected also showed that 12 (6.5%) patients had bladder stones on ultrasound examination, out of which 4 (33.3%) were from Grade I, 2 (16.7%) from Grade II, 5 (41.7%) from Grade III, and 1 (8.3%) patient from Grade IV. Additionally, anatomical anomalies that could arise from chronic outflow obstruction, notably bladder diverticulum, were recorded. In our sample, 17 (9.2%) patients had bladder diverticulum, out of which 2 (11.8%) were from Grade I, 5 (29.4%) from Grade II, 3 (17.6%) from Grade III, and 8 (47.1%) patients from Grade IV. In Figure 1, a comparison of the mean post-void volumes between each grade of IPP can be seen.

As indicated in Table 2, IPP Grade IV is associated with a higher incidence of urological morbidity among patients with BPH. This is emphasized clearly, with almost two-thirds of patients developing a UTI and half of them experiencing an episode of acute urinary retention. Additionally, it can also be noted that the frequency of urological complications increases linearly with increasing IPP Grades except for surgical intervention, which showed a higher frequency in Grade II than in Grade III (Table 2).

**Table 2:** Frequency of patients (n, %) of the various urological outcomes for each IPP Grade. IPP, intravesical prostatic protrusion; UTI, urinary tract infection

	Grade I, *n* (%)	Grade II, *n* (%)	Grade III, *n* (%)	Grade IV, *n* (%)
UTI	13 (25)	30 (42)	26 (61)	13 (75)
Acute urinary retention	6 (12)	15 (21)	15 (36)	8 (50)
Surgical intervention	3 (5)	9 (13)	4 (9)	4 (25)

Moreover, chi-square tests revealed a significant association between IPP Grade and the occurrence of UTIs (*χ*^2^ = 23.46; *P* < 0.001), acute urinary retention (*χ*^2^ = 19.83; *P* < 0.005), and surgical intervention requirement (*χ*^2^ = 26.79; *P* < 0.001). Furthermore, when controlled for age and PV, multivariate analysis using logistic regression models to calculate the odds ratio (OR) showed that increasing IPP Grade is associated with an increased risk of developing UTIs, acute urinary retention, and the need for surgical intervention. The results are demonstrated in Table 3.

**Table 3:** Odds ratio (OR) of developing the various urological outcomes for each IPP Grade. IPP, intravesical prostatic protrusion; CI, confidence interval; UTI, urinary tract infection

	OR Grade I (95% CI)	OR Grade II (95% CI)	OR Grade III (95% CI)	OR Grade IV (95% CI)
UTI	0.9 (0.7-1.2)	2.5 (1.4-4.2)	4.6 (2.7-7.9)	6.8 (3.8-9.4)
Acute urinary retention	1.1 (0.8-1.7)	2.2 (1.3-3.8)	4.7 (2.6-6.8)	7.2 (4.0-9.7)
Surgical intervention	1.3 (1-1.5)	2.1 (1.8-4.3)	3.5 (2.7-5.6)	6.4 (3.6-8.3)

Overall, the statistical test results demonstrated a compelling and consistent pattern, demonstrating the clinical significance of increasing IPP Grades with increased odds of developing urological complications (Table 3). The highest-risk group of patients is IPP Grade IV, exhibiting ORs of 6.8, 7.2, and 6.4 for developing UTIs, experiencing acute urinary retention, and requiring surgical intervention, respectively.

## Discussion

The basis for undertaking this study stems from the necessity to fill a clinical gap regarding the influence of prostatic protrusion severity on urological health. Given the multifaceted nature of BPH and its substantial burden on patient well-being, there is a need to better understand the prognostic value of IPP grading in predicting adverse urological events. UTIs, acute urinary retention, and the need for surgical intervention represent significant clinical morbidities, carrying considerable implications for patient management and outcomes [8]. Consequently, illuminating the potential relationships between prostatic protrusion grading and these outcomes is poised to enhance risk stratification, treatment decision-making, and the overall care for individuals affected by BPH-related complications. Although IPP can be routinely measured during TUS imaging of patients with BPH and can be an important adjunct in clinical assessment, it is not regularly reported in routine ultrasound prostate imaging [8].

First, our findings illustrate that having a higher IPP Grade is a statistically significant factor in patients developing an episode of UTI (*P*-value < 0.001), acute urinary retention (*P*-value < 0.005), and need for surgical intervention (*P*-value < 0.001). These findings are similar to what was reported by Yoshida et al. who found that patients with the highest IPP Grades also have the highest chance of developing acute urinary retention or requiring BPH-related surgery, which led them to believe that these patients may benefit from early surgical intervention [9]. They also found that higher grades have a higher chance of failure of trial without a catheter in those who developed acute urine retention, with a sixfold higher risk of failure in those with IPP Grade II and higher [9]. However, they did not study the likelihood of the development of UTI, which, if recurrent, is also considered an indication for surgical intervention in patients with BPH [9] and was shown in our data to have a statistically significant relation with higher IPP Grades. Furthermore, concerning surgical intervention, Lee et al. reported in 2012 that significant IPP is an independent factor for predicting better postoperative outcomes of the International Prostate Symptom Score (IPSS) [10].

Second, the independent effects of prostatic protrusion grades on urological outcomes, as evidenced by the multivariate logistic regression analyses, emphasize the strength of the previously mentioned associations. When controlling for relevant clinical variables such as age and PV, the findings suggest that the severity of prostatic protrusion exerts an independent impact on the risk of UTIs, acute urinary retention, and the likelihood of requiring surgery. These findings are underlined, particularly by patients with Grade IV IPP, who had ORs of 6.8, 7.2, and 6.4 for developing UTI, experiencing acute urinary retention, and requiring surgery, respectively. This highlights the potential utility of prostatic protrusion grading as a valuable tool for prognostication and treatment decision-making, as it may facilitate the identification of patients at heightened risk of adverse urological events, particularly those necessitating surgical intervention.

These findings are corroborated by a study conducted in 2016 by Kalkanli et al., where they found that increased IPP values are associated with lower response to α-adrenoceptor-specific management. Consequently, patients with higher IPP Grades required the addition of 5-alpha reductase inhibitors, as they exhibited higher IPSS scores [11]. Concurrently, the findings of our study align with the prospective study conducted by Tsai et al. in 2020, which similarly demonstrated a significant association between IPP and the occurrence of UTIs. Both studies underscore the clinical relevance of IPP in predicting UTIs. Moreover, it emphasizes the association of IPP with lower urinary tract symptoms, particularly in the context of urethral obstruction and acute urinary retention, providing further support for the relationship between IPP and urinary retention [12].

Another factor that can adversely impact the quality of life for BPH patients is the post-void volume [13]. Higher post-void volumes are associated with multiple irritative urinary symptoms, including a feeling of incomplete void, frequency, urgency, and nocturia [13-14]. Higher post-void volumes also are associated with urinary stasis within the bladder and provide a medium for UTIs and stone formations [15]. From our results, it is evident that when looking at the mean post-void volume, there is an almost linear increase in post-void volume as the IPP Grade increases. This can be one of the mechanisms in which higher IPP Grades contribute to the higher odds of developing certain clinical outcomes such as UTIs, bladder stones, and even bladder trabeculations or diverticulum [15].

A limitation of our study is that these assessments were conducted retrospectively by a single radiologist. However, it is important to note that the images themselves were captured by multiple radiologists using more than one ultrasound machine, introducing potential variability in the readings. In addition to that, an episode of UTI was considered by assessing patients’ history along with a positive urine routine or culture result. However, if patients had an episode of UTI that was treated by a facility outside our center, it was not recorded. Additionally, a urodynamics and uroflow setup was not available in our facility, limiting the full aspect of studying the effect of different grades on proper urinary flow. When it comes to assessing the severity of IPP, urodynamics can play a crucial role, particularly as this test measures the rate at which urine flows during urination and the maximum urine velocity (*Q*_max_) [16-17]. Given that IPP can impact the flow of urine, this test can reveal further information regarding the obstructive patterns related to protrusion severity and should be added to future studies regarding IPP.

## Conclusions

Finally, the results provide compelling evidence of the adverse relationships between higher grades of IPP and worsening urological outcomes and patient morbidity. Hence, we recommend further studies be conducted on the clinical effects of IPP and that these measurements should be considered as part of routine ultrasound prostate imaging to aid in the management of BPH cases.
